# Synergistic Anticancer Effects of Vitamin D and Plant-Derived Compounds: Molecular Mechanisms, Therapeutic Potential, and Nanotechnology-Enabled Delivery Approaches

**DOI:** 10.3390/ijms27052507

**Published:** 2026-03-09

**Authors:** Arik Dahan, Sapir Ifrah, Ludmila Yarmolinsky, Boris Khalfin, Sigal Fleisher-Berkovich, Shimon Ben-Shabat

**Affiliations:** Department of Clinical Pharmacology, Faculty of Health Sciences, Ben-Gurion University of the Negev, Beer-Sheva 8410501, Israel; sapirgar@post.bgu.ac.il (S.I.); yludmila@post.bgu.ac.il (L.Y.); boriskh83@gmail.com (B.K.); fleisher@bgu.ac.il (S.F.-B.)

**Keywords:** vitamin D, plant-derived compounds, phytochemicals, synergism, cancer

## Abstract

Vitamin D is widely recognized for its pivotal role in the prevention and treatment of various cancers. The active compounds derived from plants have garnered significant attention due to their multi-faceted anticancer properties. Given the complexity and heterogeneity of cancer, monotherapies often fall short in effectiveness. As a result, combinatorial pharmacological strategies, which utilize multiple drug agents, are increasingly being employed globally. Notably, emerging evidence highlights the potent synergistic anticancer effects of vitamin D in combination with certain phytochemicals against a variety of cancers. This review explores the cooperative mechanisms through which vitamin D and phytochemicals enhance cancer prevention and therapy. In addition to examining their synergistic effects, this review also discusses recent advancements in nanotechnology-based delivery systems for vitamin D, which hold promise for optimizing its therapeutic potential. Collectively, these findings underscore the potential of combining vitamin D with phytochemicals and innovative delivery methods as a promising strategy in the fight against cancer, paving the way for more effective, multi-targeted therapeutic approaches.

## 1. Introduction

Despite significant advances in our understanding of vitamin D and its medicinal applications, its full role in numerous biological processes remains incompletely understood. More than a decade ago, we published a review examining the biological activities of vitamin D and its analogues, with particular emphasis on bone metabolism, diabetes, cancer, skin diseases, and delivery and targeting strategies [1]. Since that time, a growing number of studies have focused on the anticancer properties of vitamin D [2], driven in part by the continued recognition of cancer as a leading cause of mortality worldwide [3]. This growing recognition has spurred further investigation into the potential of vitamin D as an anticancer agent.

However, cancer is a complex, heterogeneous disease with diverse etiologies, and conventional monotherapies often exhibit limited efficacy in its treatment and prevention [4]. Numerous experimental studies, both in vitro and in vivo, suggest that combining vitamin D with other therapeutic agents can enhance its anticancer effects. For instance, the antibiotic salinomycin is a polyether ionophore of natural origin with anticancer properties against many cancer cell lines [5]. Vitamin D_3_ and salinomycin synergy resulted in inhibiting the proliferation of MCF-7 breast carcinoma cells; a possible mechanism is endoplasmic reticulum stress transcriptional upregulation of genes (ATF6, GADD153, GADD45G, EIF2AK3, and HSPA5), which are related to this stress [6]. Combining vitamin D with repurposed drugs such as metformin and auranofin has demonstrated enhanced efficacy compared to monotherapy [7]. Another interesting example is cisplatin (a chemotherapeutic drug). The combination of vitamin D and this drug provides a better anti-proliferative property and is less toxic than cisplatin alone in the T24 bladder cancer cell line [8]. The interaction between Vitamin D and arsenic trioxide (chemotherapeutic drug) was estimated for acute promyelocytic leukemia in vitro; isobologram analysis showed a strong synergistic effect [9].

The prospect of combining vitamin D with plant-derived bioactive compounds has become particularly intriguing. Several plant extracts have been shown to enhance the anticancer effects of vitamin D [10,11]. One key advantage of such combinations is that they may offer greater efficacy and lower toxicity compared to traditional anticancer therapies. Plants are a rich source of compounds with well-established anticancer properties, including alkaloids, polyphenols (e.g., phenolic acids, stilbenes, lignans, and flavonoids), carotenoids, terpenoids, quinones, saponins, polysaccharides, and peptides. These bioactive compounds exhibit various pharmacological effects such as anti-inflammatory, antioxidant, and antidiabetic activities, all of which are critical for cancer prevention and treatment [12]. In fact, over 60% of the anticancer drugs currently in use were developed from plant sources [13]. Moreover, many plant-derived compounds are readily available, cost-effective, and less toxic than conventional anticancer agents. Their anticancer activity can partially be attributed to their antioxidant properties, as oxidative stress caused by an imbalance between reactive oxygen species (ROS) and antioxidant defenses is closely linked to cancer and other diseases [14]. ROS, including superoxide, hydrogen peroxide, hydroxyl radicals, and singlet oxygen, are highly reactive molecules that can damage cellular structures, leading to carcinogenesis [15]. Environmental stressors, such as radiation, pollution, and xenobiotics, contribute to ROS overproduction, further complicating cancer pathogenesis [16].

ROS exert a well-recognized dual role in cancer biology. At moderate levels, ROS function as pro-tumorigenic signaling molecules that promote genomic instability, activate oncogenic pathways, and support metabolic adaptations favorable to tumor growth [17,18]. Conversely, when ROS levels exceed the antioxidant buffering capacity of the cell, they induce oxidative damage and trigger cell death pathways, including apoptosis, thereby exerting tumor-suppressive effects [19]. This balance between pro-survival and pro-apoptotic ROS underscores the importance of cellular redox context, tumor type, and microenvironmental conditions in determining the net biological outcome of ROS signaling.

Phytochemicals with antioxidant activity may mitigate the formation of free radicals, regulate ROS-scavenging enzymes, and influence numerous cellular processes by modulating various molecular targets and signaling pathways [20,21]. These pathways are involved in vital biological processes, including cell survival, proliferation, apoptosis, migration, angiogenesis, hormonal regulation, and immune responses [22].

A comprehensive analysis of scientific literature reveals substantial overlaps in the anticancer mechanisms of vitamin D and plant-derived bioactive compounds. Although the synergistic anticancer effects of vitamin D and phytochemicals represent a promising strategy for cancer prevention and treatment, several challenges—including limited bioavailability, stability, and targeted delivery—have hindered their clinical translation. This review addresses these limitations by integrating current evidence on shared molecular pathways, synergistic therapeutic effects, and recent advances in delivery strategies, with particular emphasis on nanotechnology-based systems designed to enhance the efficacy and safety of vitamin D. Collectively, after many years of scarce attention [23], this work aims to provide an updated and integrated perspective on the role of vitamin D in cancer prevention and therapy, its delivery approaches, and the therapeutic potential of its synergistic interactions with phytochemicals.

## 2. Overview of the Anticancer Activity of Vitamin D

**Figure 1:**
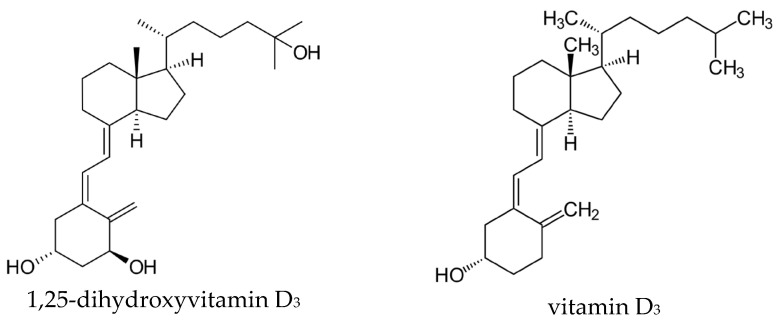
Molecular structure of 1,25-dihydroxyvitamin D_3_ and vitamin D_3_.

Vitamin D exists in several vitamers. Figure 1 demonstrates the most active vitamers, 1,25-dihydroxyvitamin D_3_ and vitamin D_3_. Vitamin D_1_ is known as a mixture of ergocalciferol and lumisterol. Vitamin D_2_ is ergocalciferol, vitamin D_3_ is cholecalciferol, vitamin D_4_ is 22-dihydroergocalciferol and vitamin D_5_ (sitocalciferol), with vitamin D_2_ and D_3_ being the most biologically relevant and widely studied forms [1,24]. Vitamin D is synthesized in the skin from 7-dehydrocholesterol upon exposure to ultraviolet radiation and can also be obtained from dietary sources or supplementation [25]. Its biological effects are mediated primarily through binding to the vitamin D receptor (VDR), which activates both genomic and non-genomic signaling pathways [26]. VDRs are expressed in a broad range of tissues and cell types, including skin, skeletal muscle, adipose tissue, pancreatic endocrine cells, immune cells, and vascular endothelium [27]. Although VDR activation has been reported to promote certain cancer types under specific conditions [28], it is more commonly associated with protective effects, particularly in maintaining cellular redox balance and limiting excessive ROS production [29].

Genomic vitamin D signaling involves the formation of a heterodimer between the ligand-activated VDR and the retinoid X receptor (RXR). This complex binds to vitamin D response elements within target genes, leading to transcriptional modulation [30]. These genomic effects develop over hours to days and regulate a wide array of biological processes, including innate and adaptive immunity, energy metabolism, cell proliferation and differentiation, calcium–phosphate homeostasis, and neuroprotection [31,32]. Importantly, the expression of CYP24A1, an enzyme responsible for vitamin D catabolism into inactive calcitroic acid, has emerged as a predictive marker of vitamin D efficacy in cancer patients and is frequently upregulated in tumor cells [33].

In contrast, non-genomic vitamin D signaling occurs independently of direct gene transcription and involves rapid activation of intracellular signaling cascades [31,32]. Cancer progression is closely linked to VDR-mediated signaling [34], and accumulating evidence suggests that vitamin D can exert anticancer effects even in the absence of functional VDRs, indicating the involvement of alternative pathways [35].

**Figure 2:**
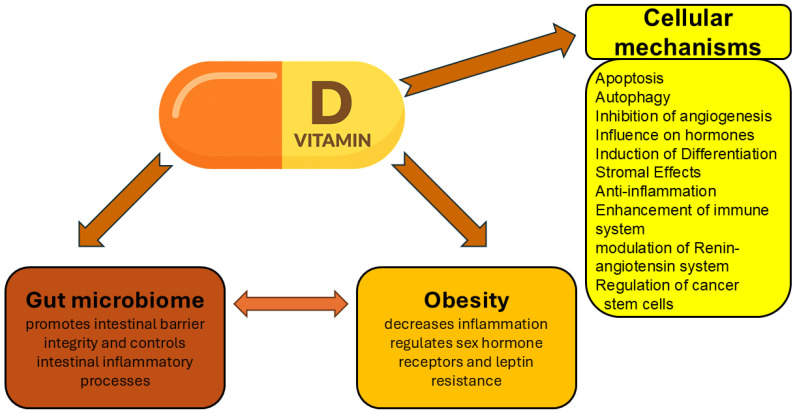
Mechanisms of anticancer action of vitamin D. The influence of vitamin D on gut microbiome, obesity and various cellular mechanisms.

Clinical and epidemiological studies consistently associate vitamin D deficiency with an increased risk of multiple malignancies. Low serum vitamin D levels have been correlated with a higher incidence of colorectal, ovarian, lung, breast, and prostate cancers [36]. At the cellular level, vitamin D regulates numerous interconnected processes (Figure 2), including the stabilization of calcium signaling and redox homeostasis, thereby limiting ROS-mediated cellular damage [37].

Vitamin D exerts multiple anticancer effects, including the suppression of tumor cell proliferation [38,39], induction of apoptosis [40], modulation of autophagy [41], suppression of angiogenesis [42], regulation of hormone signaling [43], promotion of cellular differentiation, inhibition of epithelial-to-mesenchymal transition [44], antagonism of Wnt/β-catenin signaling [45], stromal remodeling [46], regulation of cancer stem cells [47], enhancement of antitumor immunity [48], anti-inflammatory effects [49], modulation of the gut microbiome [50], regulation of the renin–angiotensin system [51], and protection against oxidative stress-induced DNA damage [52].

Among these mechanisms, inhibition of cancer cell proliferation is partly mediated by the ability of vitamin D to suppress the Warburg effect, characterized by enhanced aerobic glycolysis, which is a hallmark of many cancer types [53]. Vitamin D also induces apoptosis through cancer-type-specific mechanisms, regulating key apoptotic mediators such as caspases and Bcl-2 family proteins [54]. For example, vitamin D upregulates caspase activity in non-malignant MCF-12A cells but not in malignant MCF-7 breast cancer cells, highlighting context-dependent responses [55].

Autophagy, a tightly regulated self-degradative process controlled by autophagy-related genes (ATGs), plays a dual role in cancer progression and therapy. The process involves phagophore initiation, autophagosome formation, fusion with lysosomes, and cargo degradation [56]. Vitamin D has been reported to modulate autophagy, contributing to its therapeutic efficacy in cancer treatment [57].

Angiogenesis, the formation of new blood vessels, is essential for tumor growth and metastasis [58]. Several in vivo studies have demonstrated that vitamin D can suppress angiogenesis, thereby limiting tumor progression [59,60,61]. In addition, vitamin D exerts immunomodulatory effects by activating immune cells, inducing pro-apoptotic proteins [62], and upregulating cyclin-dependent kinase inhibitors [63]. Notably, VDR is expressed in nearly all leukocyte populations, including macrophages, dendritic cells, activated CD4^+^ and CD8^+^ T cells, and B cells, underscoring its role in immune regulation [64].

Vitamin D also interacts with key oncogenic signaling pathways, including Wnt/β-catenin, NF-κB, PI3K/Akt, and p53, thereby influencing tumor growth, invasion, and metastasis [65]. Beyond direct cellular effects, vitamin D contributes to cancer prevention through modulation of the gut microbiome, enhancement of intestinal barrier integrity, and regulation of intestinal inflammation [66]. Vitamin D has a positive effect on the gut microbiota; enhances the growth of beneficial bacteria such as *Ruminococcaceae*, *Akkermansia*, *Faecalibacterium* and *Coprococcus*; and suppresses *Firmicutes* (*Staphylococcus aureus*, *Streptococcus pneumoniae*, *Listeria* and *Bacillus anthracis*) activity [67].

Obesity is a recognized risk factor for cancer incidence and mortality, particularly in breast and colorectal cancers [68]. Chronic low-grade inflammation associated with obesity promotes carcinogenesis and tumor progression [69]. Vitamin D may mitigate these pro-carcinogenic effects by reducing inflammation [70], regulating sex hormone receptor signaling, and improving leptin sensitivity, thereby exerting protective metabolic and immunological effects [71].

## 3. Synergy Between Vitamin D and Phytochemicals

Many phytochemicals possess intrinsic anticancer properties capable of preventing tumor initiation and inhibiting cancer cell proliferation [72]. Figure 2 provides the main mechanisms of anticancer action of vitamin D. Anticancer compounds of plant origin not only encompass all modes of action of vitamin D (Figure 2) but also take actions on multiple signaling pathways and have various mechanisms that are not characteristic of vitamin D [73]. This is also true with regard to several phytochemicals in the context of breast cancer [74]. Other examples are polyphenols that may modulate signal transduction pathways and induce apoptosis [75] even as alkaloids such as vincristine, vinblastine, colchicine, vindesine and vincamine disturb mitotic spindle assembly and consequently break cell division [76]. As already mentioned, obesity is one of the most prominent factors driving the development of cancer. Many compounds of plant origin may be beneficial in obesity management, and molecular mechanisms of their activities include adipose tissue mass reduction by increasing fat cell apoptosis, hindering precursor cell proliferation, regulation of lipid metabolism, and enhancement of energy expenditure; inhibiting triglyceride absorption by decreasing pancreatic lipase and suppressing appetite [77].

When combined with vitamin D, these plant-derived compounds can potentiate their anticancer effects through multiple mechanisms, including modulation of ROS-scavenging enzymes, inhibition of cell cycle progression, induction of apoptosis and autophagy, suppression of angiogenesis, and inhibition of metastasis. Vitamin D and phytochemicals share overlapping therapeutic targets, and certain combinations demonstrate synergistic anticancer activity.

Experimental studies suggest that some phytochemicals interact directly with the vitamin D receptor [78]. For instance, the alkaloids coclaurine and reticuline upregulated VDR expression and inhibited colorectal cancer progression in vitro [79]. Similarly, vitexin was shown to target VDR and modulate macrophage polarization, thereby preventing colorectal cancer [80], while asperuloside enhanced colonic VDR expression, alleviating colitis symptoms and reducing tumor burden [81]. Isoflavones such as genistein, biochanin A, and formononetin suppressed CYP24A1 induction in Huh7 cancer cells under both normoxic and hypoxic conditions [82]. These findings suggest that additional plant-derived compounds capable of direct VDR binding remain to be discovered and characterized.

Combination therapies involving vitamin D and phytochemicals have shown promising results, particularly because they target common apoptotic and cell survival pathways. Original studies evaluating these synergistic interactions are summarized in Table 1, with phytochemicals listed alphabetically. However, the anticancer potential of many phytochemicals has not been studied in sufficient depth to allow direct comparison with vitamin D. Among well-characterized compounds, the polyphenol resveratrol has been extensively investigated and serves as a model for comparison. Resveratrol, found in wine, chocolate, cocoa, and various fruits and juices [83], exhibits broad anticancer activity, functioning as both a chemopreventive [84] and therapeutic agent [85].

Vitamin D and resveratrol share numerous mechanistic similarities. Both regulate critical processes in cell proliferation, differentiation, and apoptosis [86] and modulate key signaling molecules, including NF-κB, Akt, MAPK, Fas antigen receptors, TNF-α, Bcl-2, Bcl-xL, and p53 [87]. Glutathione has been shown to mediate the anticarcinogenic effects of both resveratrol [88] and vitamin D [89]. The superoxide dismutase family is important for the reduction in deleterious effects of reactive oxygen species [90]. Regulation of the enzyme superoxide dismutase is performed by both vitamin D [91] and resveratrol [92]. Both agents influence caspase-3 activity, an effector of apoptotic pathways [93,94]. Similarly to vitamin D [95], resveratrol activates protein kinases involved in cell proliferation, differentiation, apoptosis, and malignant transformation [96]. Furthermore, vitamin D and resveratrol inhibit tumor angiogenesis through downregulation of vascular endothelial growth factor (VEGF) [97,98,99]. The combination of vitamin D with flavonoids has also been associated with reduced risk of obesity-related breast cancer [100].

As mentioned throughout the paper, since cancer is so complex in its etiology, combining vitamin D with other active agents is effective but only at a specific concentration and ratio. The mechanism responsible for synergistic antioxidant activity has not been explained perfectly yet due to the challenging nature of the phytochemicals. The problem of anticancer effect in a mixture of vitamin D and plant compounds is very difficult to solve because it depends on the chemical structure of the compounds, their concentrations and molecular ratio, applied solvent, the reaction time and treatment of the sample [101].

Isobologram analysis gives the possibility to estimate the interaction between two or more drugs as synergistic, additive, or antagonistic mathematically based on the combination index [102]. Thus far, only a few studies have been undertaken to evaluate the synergism of vitamin D with some drugs [103,104], but not with phytochemicals.

Combinations of vitamin D and plant active components were not used in clinical trials. Although these combinations mediate their anticancer effects by modulating multiple signaling pathways, many challenges are connected with their inadequate bioavailability, pharmacokinetics, metabolism and toxicity. One of the important obstacles to successful clinical feasibility is a lack of pharmacodynamic biomarkers allowing to estimate anticancer effect in a proper form [105].

**Table 1:** Vitamin D_3_ and phytochemicals synergistically contribute to anticancer effect.

Compound and Source	Structure	Cancer Model	Mechanism	References
β-carotene Fungi, plants, fruits	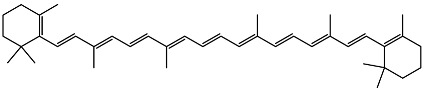	Esophageal cancer in vitro	Induction of apoptosis in EC9706 cells	[106]
Aglycon Agave species	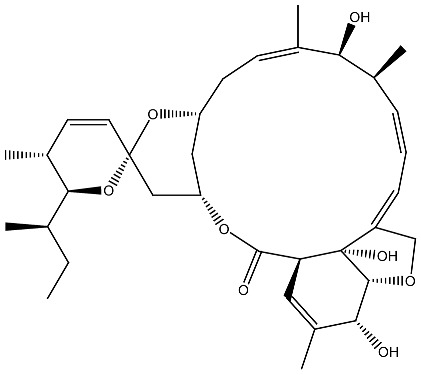	Prostate cancer in vitro	Synergistically decreases cell viability and proliferation.	[107,108]
Arsantin *Artemisia santolina*	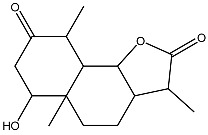	Leukemia in vitro	HL-60 cells differentiation	[109]
Capsaicin Chili peppers	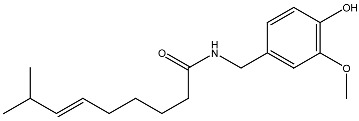	Leukemia in vitro	HL-60 cells differentiation	[110]
Carnosic acid Rosemary, common sage	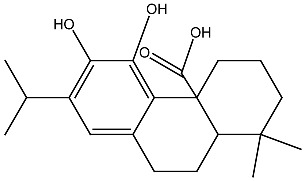	Leukemia in vitro	Inhibits proliferation and augments differentiation of human leukemic cells	[111]
Costunolide *Saussurea costus*	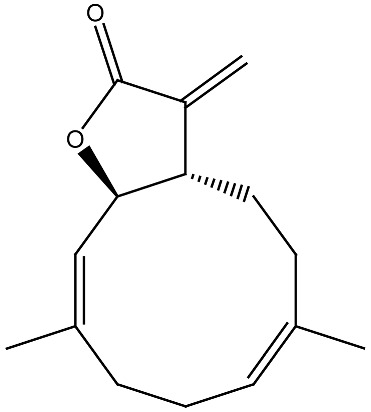	Leukemia in vitro	HL-60 leukemia cell differentiation	[112]
Cotylenin A *Cladosporium* spp.	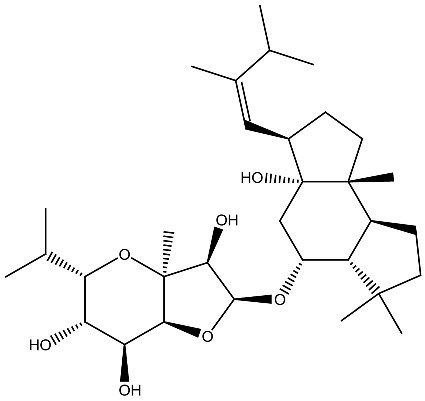	AML in vitro	Inducing the monocytic differentiation of AML cells	[113]
Curcumin *Curcuma longa*	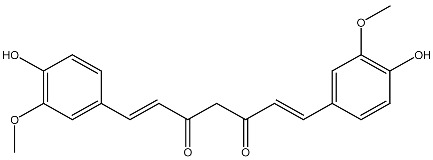	Prostate cancer, leukemia in vitro	Inhibition of proliferation of HL-60 cells	[114]
			Effect and inhibitory metastatic characteristics in prostate cancer cells	[115]
Epigallocatechin Green tea	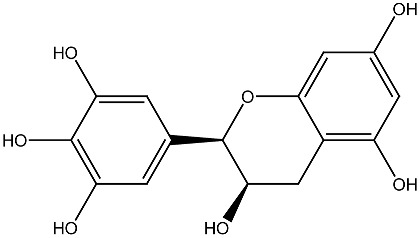	Uterine myoma Clinical research	The total myoma volume decreased by 34.7%	[116]
Eugenol Clove oil	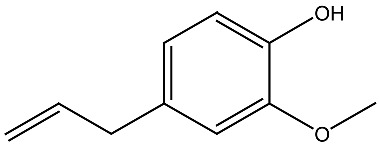	Leukemia in vitro	Inhibition of proliferation of HL-60 cells	[117]
Genistein Soybeans	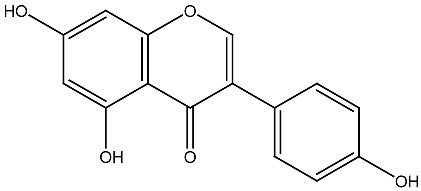	Prostate cancer in vitro	Inhibition of proliferation of 1,25(OH)_2_D_3_ in DU145 cells	[118]
Ginsenoside Rg3 *Panax ginseng*	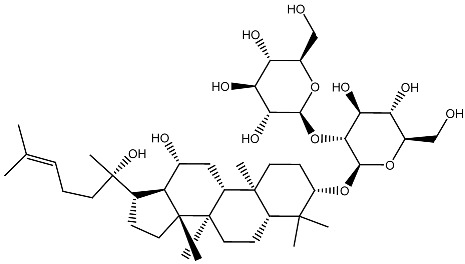	Prostate cancer in vivo and in vitro	Induces apoptosis and reduces cell proliferation	[119]
Ginsenoside Rh2 *Panax ginseng*	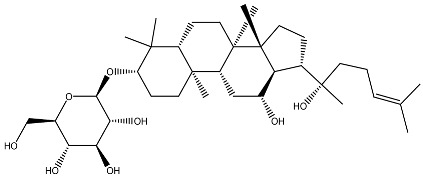	Prostate cancer in vitro	Stimulating apoptosis and reduced cell proliferation	[120]
Glycyrrhizin *Glycyrrhiza* *glabra*	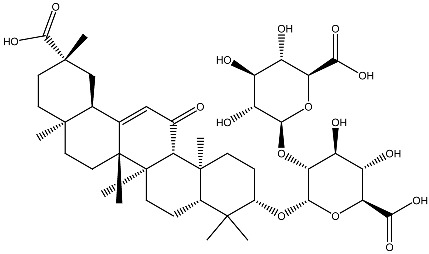	Prostate cancer in vivo and in vitro	Inhibits growth of LNCaP and C4-2 PCa cells	[107,121]
Humulone *Humulus lupulus*	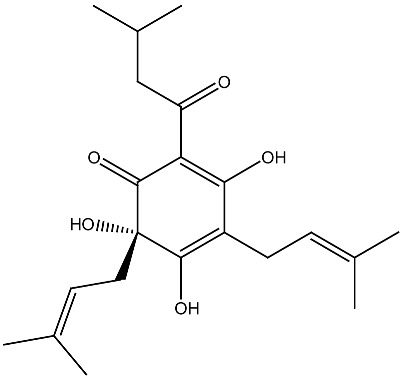	Leukemia in vitro	HL-60 cells differentiation	[122]
Lycopene Tomatoes, vegetables and fruits	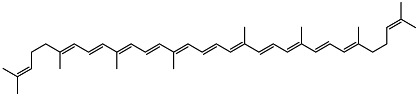	Leukemia in vitro	Inhibition of cell cycle progression and induction of differentiation in HL-60 cells	[123]
Neferine Green seed embryo of the lotus plant	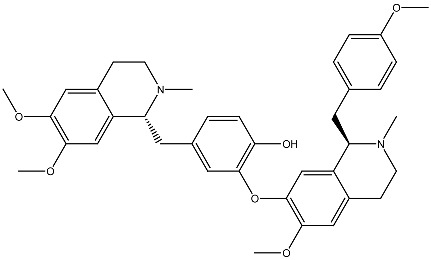	Colorectal cancer in vitro	Inhibition of metastasis	[124]
Parthenolide *Tanacetum* spp.	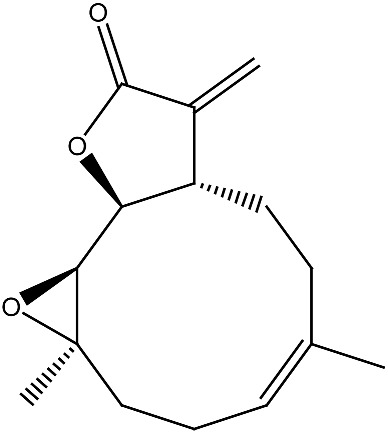	Leukemia in vitro	Induced HL-60 cell differentiation into monocytes via the inhibition of NF-κB activity	[125]
Quercetin Many plants	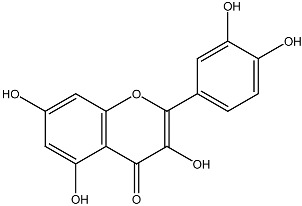	Breast cancer in vitro and in vivo	Inhibition of cell cycle progression	[126]
Resveratrol Grape, berries, peanuts	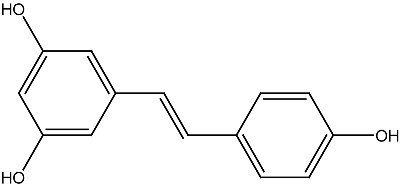	Lung cancer in vitro	Influence on cell proliferation, inhibition, cell cycle, apoptosis, and expression of cytokines and proteins. Affect VDR and other nuclear receptors indirectly	[127,128]
Silibinin *Silybum* *marianum*	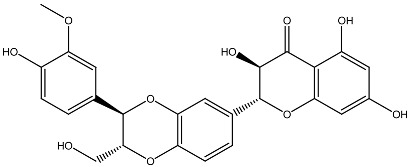	AML in vitro	Anti-proliferative and anti-migratory effects Differentiation of AML Cells	[129,130]
Sulforaphane Cruciferous vegetables	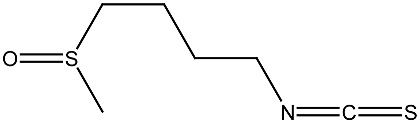	Prostate cancer in vitro and in vivo	Modulate the JNK/MAPK signaling pathway and suppress intestinal Wnt-signaling and tumorigenesis in obese mice.	[131,132]
Thymoquinone *Nigella sativa*	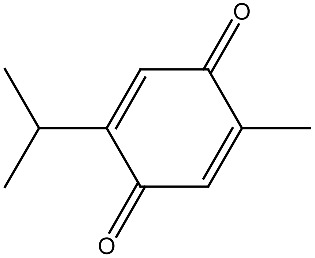	Colon cancer in vitro	Anti-tumorigenic effects	[133]
Yomogin *Artemisia iwayomogi*	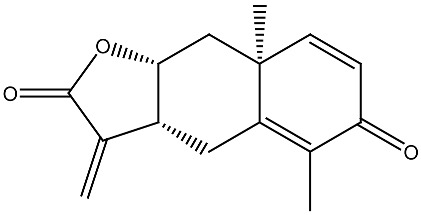	Leukemia in vitro	Differentiation of HL-60 cells	[134]

## 4. Co-Delivery Systems for the Prevention and Treatment of Cancer

### 4.1. Importance of Co-Delivery Systems

Co-delivery systems are spectacular strategies because of the opportunity to achieve synergistic therapeutic effects, which are more effective in overcoming the multidrug resistance in many cancer cells. These combined therapies can provide better outcomes than separate delivery approaches carrying either phytochemicals or vitamin D. Liposomes, emulsions and nanosystems are widespread delivery systems for vitamin D and phytochemicals [135].

The synergistic effect of vitamin D and phytochemicals relates to various biological mechanisms that enhance their combined impact, for example, their molecular targets, enzymatic pathways and so on.

Herein, designing novel nano-scale platforms to deliver vitamin D and anticancer active compounds of plant origin is also discussed.

### 4.2. Approaches to Vitamin D Delivery

Although the anticancer properties of vitamin D have been extensively documented in the prevention and treatment of colorectal, prostate, and breast cancers [136,137], significant challenges continue to limit its clinical application. Administration of vitamin D at high doses is often ineffective and may lead to adverse effects such as hypervitaminosis D, hypercalcemia, and hyperphosphatemia. Moreover, serious side effects have been reported when vitamin D is combined with other anticancer drugs in cancer patients [121]. Consequently, the therapeutic and chemopreventive potential of vitamin D has not yet been fully realized in clinical oncology. An additional limitation is its relatively short half-life in the bloodstream, which further restricts sustained therapeutic efficacy [138].

**Figure 3:**
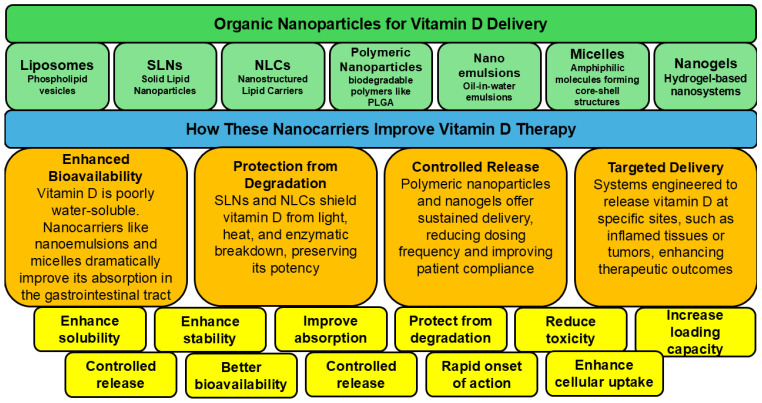
The targeted delivery of vitamin D. The image depicts various organic nanoparticles used for vitamin D delivery and their properties.

Globally, a substantial proportion of the population fails to meet the recommended daily vitamin D intake due to insufficient sunlight exposure [139]. Furthermore, vitamin D is highly susceptible to degradation during storage and food processing, and upon exposure to heat, light, and moisture, resulting in reduced stability and bioavailability [140]. Its poor water solubility and limited intestinal absorption further compromise its anticancer effectiveness. To address these limitations, targeted delivery strategies have emerged as a promising approach to enhance vitamin D bioavailability and therapeutic performance. As illustrated in Figure 3, advanced delivery systems including nanoencapsulation, nanoemulsions, complexation, mixed micelle systems, conjugation strategies, carbon quantum dots, and crosslinking techniques have been explored to overcome these barriers [141,142]. Notably, such formulations have been shown to enhance vitamin D bioavailability by two- to eight-fold, with encapsulation techniques significantly improving its bioaccessibility [143,144]. A recent review is devoted to the targeted delivery of vitamins in the context of gastrointestinal disorders, including colorectal cancer [145].

Encapsulation strategies offer additional advantages by protecting vitamin D from environmental degradation and improving its stability under adverse conditions [146]. In vivo studies have demonstrated that encapsulated vitamin D improves key physiological parameters, including circulating vitamin D levels, intestinal calcium and phosphorus absorption, and glycemic control in experimental models [147]. For example, vitamin D_3_ encapsulated in gum Arabic exhibited superior absorption and bioavailability compared with its free form [148]. While nanoemulsions based on gum Arabic have been developed for vitamin D delivery, further investigation of their physicochemical properties, stability, and biological activity remains necessary [149]. Alternative approaches include the use of non-toxic, long-term Pickering emulsions based on zein–chitosan complex particles, which have shown promise for the encapsulation and sustained delivery of vitamin D_3_ [150]. The development of a complex nanoemulsion by combining pea protein and Tween 80 together gave the possibility to enhance vitamin D uptake and its transport efficiency significantly [151].

Targeted nanocarrier systems have also been explored to enhance the anticancer efficacy of vitamin D in drug-resistant tumors. Epidermal growth factor receptor (EGFR)-targeted liposomal nanoparticles co-encapsulating vitamin D and the 24-sulfoxamine derivative of 1,25-dihydroxyvitamin D_3_ (CTA091) demonstrated improved cellular uptake and more effective inhibition of colony formation in drug-resistant lung cancer models compared with free compounds [152]. Similarly, targeted delivery of vitamin D-loaded nanoparticles to drug-resistant C6 glioma cells significantly reduced cell proliferation and increased sensitivity to chemotherapeutic agents such as doxorubicin, epirubicin, and docetaxel (*p* < 0.05) [153]. Importantly, evidence suggests that targeted vitamin D delivery is more effective during early stages of carcinogenesis than in advanced disease [154].

Carbon Nanofiber–Sodium Alginate Composite Aerogels effectively delivered vitamin D to human colorectal cancer cells, improved their bioavailability, and significantly decreased cancer cell proliferation and migration [155].

Combination delivery systems have further expanded the therapeutic potential of vitamin D. Sodium caseinate micelles co-loaded with vitamin D and etoposide exhibited enhanced anticancer activity against human MDA-MB-231 and MCF-7 breast cancer cell lines compared with free vitamin D [156]. Similarly, micellar formulations co-encapsulating paclitaxel and vitamin D have been developed for intravenous or intratumoral administration in pancreatic cancer models, demonstrating improved therapeutic outcomes [157].

The proliferation of melanoma cells (B16 F10 cell line) was suppressed by liposomal vitamin D and free vitamin D; however, the former had a better antiproliferative effect and the most pronounced decrease in the expression of AKT1, BCL2, and PI3K genes [158].

In addition, nanoliposomal encapsulation of vitamin D using thin-film hydration and sonication methods has been shown to markedly enhance its solubility, cellular uptake in Caco-2 cells, storage stability (even at 25 °C), and stability in aqueous and gastrointestinal environments [159]. Collectively, these advances highlight the critical role of innovative delivery systems in overcoming the physicochemical and biological limitations of vitamin D, thereby unlocking its full potential as an anticancer agent.

### 4.3. Lymphatic Transport of Vitamin D

The lymphatic system harbors numerous potential therapeutic targets that may influence various types of cancer [160]. For drugs to be effectively transported via the lymphatic route, they must exhibit significant lipophilicity. Specifically, a log *p* value greater than 5 and triglyceride solubility exceeding 50 mg/mL have been suggested as critical physicochemical thresholds for efficient lymphatic transport. Vitamin D_3_, known for its high lipophilicity (with a log *p* value around 9), relies on this pathway for its oral bioavailability [161].

**Figure 4:**
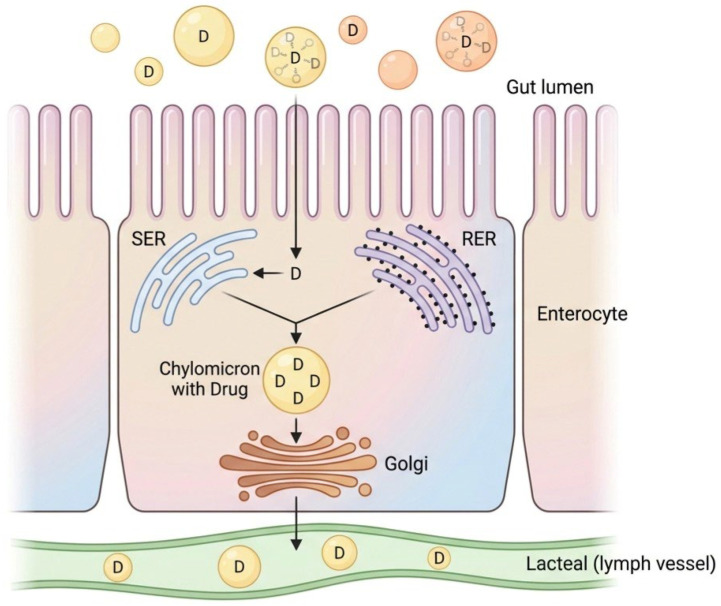
Lymphatic transport of vitamin D. The three events in the sequential assembly of chylomicrons within the enterocyte are presented: (1) formation of primordial lipoproteins in the rough endoplasmic reticulum (RER); (2) synthesis of large triglyceride-rich droplets in the smooth endoplasmic reticulum (SER); (3) biosynthesis of chylomicrons in the core expansion process. It was demonstrated that most of the dose (75%) of lipophilic vitamin D gains access to the lymphatic system by incorporating into the lipidic core of the chylomicrons.

As illustrated in Figure 4, the sequential assembly of chylomicrons within enterocytes involves several key steps: the formation of primordial lipoproteins in the rough endoplasmic reticulum (RER), the synthesis of large triglyceride-rich droplets in the smooth endoplasmic reticulum (SER), and the subsequent biosynthesis of chylomicrons through core expansion.

Lymphatic transport of vitamin D is particularly critical for preventing metastasis, as the lymphatic system plays an essential role in the metastatic dissemination of cancer cells [162].

### 4.4. Formulations Combining Vitamin D with Plant-Derived Compounds

A major limitation in the therapeutic use of phytochemicals lies in their inherent properties, including low bioavailability due to rapid metabolism, poor cellular uptake, and inadequate targeting [163]. These factors restrict their effectiveness, particularly when combined with vitamin D for cancer treatment. This highlights the critical need for the development of innovative drug delivery systems that enhance the pharmacokinetic profile, cellular uptake, and therapeutic efficacy of vitamin D in combination with plant-derived compounds.

Several phytochemicals have been identified as bioenhancers, known to improve the solubility and bioavailability of vitamin D [164]. However, the mechanisms underlying their bioenhancing effects remain poorly understood, and research into their modes of action is limited. Despite the importance of co-delivery systems, relatively few studies have investigated formulations combining vitamin D with phytochemicals. One such study explored the co-encapsulation of vitamin D and rutin in chitosan–zein microparticles using hydrophobic interactions, hydrogen bonding, Van der Waals forces, and nonspecific electrostatic neutralization between the oppositely charged polymers [165,166].

In addition, curcumin and vitamin D have been co-encapsulated in nanoliposomes [167] and nanoemulsions [168], resulting in enhanced solubility, stability, and bioavailability compared to their free forms. Similarly, cinnamon essential oil, which contains several anticancer compounds such as monoterpenes and terpenoids [169], has been studied in combination with vitamin D, though not extensively. A recent study demonstrated that a vitamin D-encapsulated cinnamon oil nanoemulsion induced G0/G1 cell cycle arrest in A549 lung carcinoma cells, increased the expression of pro-apoptotic markers (Bax, caspase-3, and caspase-9), and decreased anti-apoptotic Bcl-2 levels [170]. This highlights the significant potential of combined vitamin D–phytochemical formulations while also emphasizing the substantial gaps in current knowledge that necessitate further investigation.

In experimental in vitro studies, micellization significantly enhanced the uptake of vitamin D by buccal and intestinal cells. While curcuma extract further improved the uptake of micellated vitamin D, its bioefficacy remained unchanged [171].

The health benefits of vegetables are well-documented, and the concept of using vegetable-derived systems for vitamin D delivery is particularly intriguing. Notably, a study demonstrated that vitamin D-loaded tomato-derived extracellular vesicles effectively reduced the growth, spread, and survival of colon cancer cells [172]. This innovative approach underscores the potential of plant-based delivery systems in enhancing the therapeutic effects of vitamin D in cancer treatment.

## 5. Conclusions

The synergistic interactions between vitamin D and phytochemicals hold considerable promise for cancer prevention and therapy by modulating multiple cellular and molecular pathways. However, the majority of potential combinations remain largely unexplored, representing a significant opportunity for future research.

To fully realize this therapeutic potential, future studies should prioritize the identification of novel, effective vitamin D–phytochemical combinations and the development of advanced drug delivery systems to enhance bioavailability and stability. Unfortunately, the effect of various factors on the bioavailability of vitamin D was not estimated in any clinical studies [173]. Moreover, there is a pressing need for comprehensive in vivo studies and well-designed clinical trials to establish standardized protocols and evaluate the efficacy of these combinations in cancer treatment. Addressing these challenges will be critical for translating preclinical findings into practical, safe, and effective anticancer strategies.

## Data Availability

No new data were created or analyzed in this study. Data sharing is not applicable to this article.

## Abbreviations

The following abbreviations are used in this manuscript:

Akt	Protein kinase B
AML	Acute Myeloid Leukemia
ATGs	Autophagy-related genes
BCL-2	B-cell lymphoma 2
Bcl-xL	B-cell lymphoma—extra large
CYP24A1	Cytochrome P450 family 24 subfamily A member 1
EGFR	Epidermal growth factor receptor
MAPK	Mitogen-Activated Protein Kinase
NF-κB	Nuclear Factor kappa-light-chain-enhancer of activated B cells
NLCs	Nanostructured lipid carriers
PI3K	Phosphoinositide 3-kinase
RER	Rough Endoplasmic Reticulum
ROS	Reactive oxygen species
RXR	Retinoid X receptor
SER	Smooth Endoplasmic Reticulum
SLNs	Solid Lipid Nanoparticles
TNF-α	Tumor Necrosis Factor-alpha
VDR	Vitamin D receptor
VEGF	Vascular endothelial growth factor
